# Meta-Reinforced-Model-Based Planning and Fault-Tolerant Control for a Saturation Diving Decontamination Decompression Chamber

**DOI:** 10.3390/s25113534

**Published:** 2025-06-04

**Authors:** Nan Zhang, Qijing Lin, Zhuangde Jiang

**Affiliations:** School of Mechanical Engineering, Xi’an Jiaotong University, Xi’an 710049, China

**Keywords:** saturation diving, decompression chamber, reinforcement learning, meta-learning, model-based planning, fault-tolerant control

## Abstract

Saturation diving is the only viable method that enables divers to withstand prolonged exposure to high-pressure environments, and it is increasingly used in underwater rescue and marine resource development. This study presents the control system design for a specialized saturation diving decontamination decompression chamber. As a multi-compartment structure, the system requires precise inter-cabin pressure differentials to ensure safe decontamination and ventilation control under dynamic conditions, particularly in the presence of potential faults, such as valve offset, actuator malfunction, and chamber leakage. To overcome these challenges, we propose a novel model-based planning and fault-tolerant control framework that enables adaptive responses and maintains resilient system performance. Specifically, we introduce a trajectory-planning algorithm guided by policy networks to improve planning efficiency and robustness under system uncertainty. Additionally, a meta-learning-based fault-tolerant control strategy is proposed to address system disturbances and faults. The experimental results demonstrate that the proposed approach achieves higher cumulative rewards, faster convergence, and improved robustness compared to conventional methods. This work provides an effective and adaptive control solution for human-occupied hyperbaric systems operating in safety-critical environments requiring fail-operational performance.

## 1. Introduction

To overcome the limitations of conventional and helium-based deep diving, saturation diving was introduced by George F. Bond to enable repeated excursions from a pressurized habitat, requiring only one slow controlled decompression at the end of the mission [1]. This technique greatly improves deep-sea operation safety and efficiency and has been widely adopted in various applications, such as subsea construction, resource exploration, and marine research.

As the core equipment in saturation diving systems, the technological advancement of the decompression chamber is directly linked to the safety and reliability of diving operations. In recent years, decompression chamber control systems have undergone significant development, evolving from manually operated experience-based methods to increasingly automated and intelligent approaches. Early decompression chambers largely relied on preset decompression routines and manual operations, which resulted in slow response times and limited safety. To improve the accuracy and safety of the decompression process, Yang et al. proposed a simulation-based control and monitoring framework for saturation diving decompression chambers [2]. They developed a virtual platform simulating real-world sensors and actuators and demonstrated its suitability for control system development and diver training. Gutvik et al. were the first to introduce model predictive control into the field of decompression for diving applications [3]. By using a two-phase dynamic bubble model, they optimized the diver’s decompression trajectory through real-time control algorithms that minimized total decompression time while strictly limiting venous bubble growth to mitigate the risk of decompression sickness. This method enabled the computation of optimal decompression curves in real time and supported adaptive control based on individual physiology and task demands. To address emergency scenarios, Imbert et al. analyzed the outcomes of several emergency decompression events and summarized feasible strategies, such as an initial rapid ascent, increased oxygen partial pressure, and inert gas switching, to expedite decompression [4]. Risberg et al. conducted a comparative analysis of various transfer-under-pressure decompression procedures and highlighted substantial discrepancies between decompression models and real-world practice, underscoring the need for improved model accuracy and better experimental validation in the field of decompression control and planning [5].

Recent advancements in automatic control for medical hyperbaric oxygen chambers have also provided valuable insights for decompression chamber systems. Gracia et al. developed a precise automatic pressure control system through system modeling and parameter identification for a hyperbaric oxygen chamber [6]. They implemented an RST (pole-zero cancellation) controller that enabled accurate tracking of pressure profiles and significantly enhanced control precision and system robustness. Motorga et al. introduced artificial intelligence into the control of surgical hyperbaric chambers by developing a neural-network-based adaptive PID controller [7]. This controller dynamically adjusted PID parameters through real-time learning, effectively reducing overshoot and stabilization time in pressure control while improving disturbance rejection. Their adaptive control approach demonstrated the substantial potential of AI technologies for controlling high-pressure environments.

Despite significant progress in the modeling methods, decompression optimization, and control strategies, several key challenges remain. On one hand, most existing studies focus on single-chamber control, while the complex dynamic coupling effects in multi-chamber coordination scenarios, particularly under special operating conditions, remain underexplored, and dedicated control strategies for such situations are lacking. On the other hand, most current algorithms rely on fixed models or heuristic rules and are limited in their ability to rapidly adapt to non-stationary dynamics and environmental variations. Therefore, it is essential to establish control algorithms that support accurate regulation and rapid adaptation.

Condition monitoring, which involves continuous or periodic evaluation of system performance and integrity through sensor data, has become increasingly critical to prevent unexpected failures and enhance reliability [8]. Deep learning techniques, such as physics-informed autoencoders with spatial latent spaces, have demonstrated superior capability in anomaly detection and system degradation identification, highlighting their robustness and accuracy in fault analysis [9].

This study proposes a model-based planning and fault-tolerant control framework, which offers higher data efficiency and better adaptability to environmental changes. The organization of this paper is as follows: Section 2 introduces the overall system design and control configuration of the saturation diving decontamination decompression chamber. Section 3 presents a model-based trajectory planning control algorithm, enhanced by reinforcement learning guidance to improve planning performance. Section 4 proposes a meta-learning-based method for fault-tolerant control, enabling rapid adaptation through policy updates. Section 5 evaluates the proposed algorithms, and Section 6 concludes the study.

## 2. System Design of the Saturation Diving Decompression Chamber

When divers return to a ship via the saturation diving bell, they are transferred into the decompression chamber, where transfer and decompression operations are carried out. The working pressure inside the decompression chamber corresponds to several atmospheres, which is equal to the pressure at the underwater work site. If divers carry contaminants, decontamination procedures must be completed in the transfer cabin. At this stage, the decompression chamber functions as a multi-cabin interconnected system, requiring a controlled pressure differential between compartments during operation. This pressure differential ensures that, when the doors between cabins are opened, ventilation flows in a forward direction to prevent contamination from spreading from one compartment to the next. At the same time, to allow personnel to manually open and close the inter-compartment doors and to ensure operational safety during door transitions, the pressure difference between chambers must be kept below a specified limit. This study designs the specialized saturation diving decontamination decompression chamber and its corresponding control system.

### 2.1. Overall System Design

**Figure 1:**
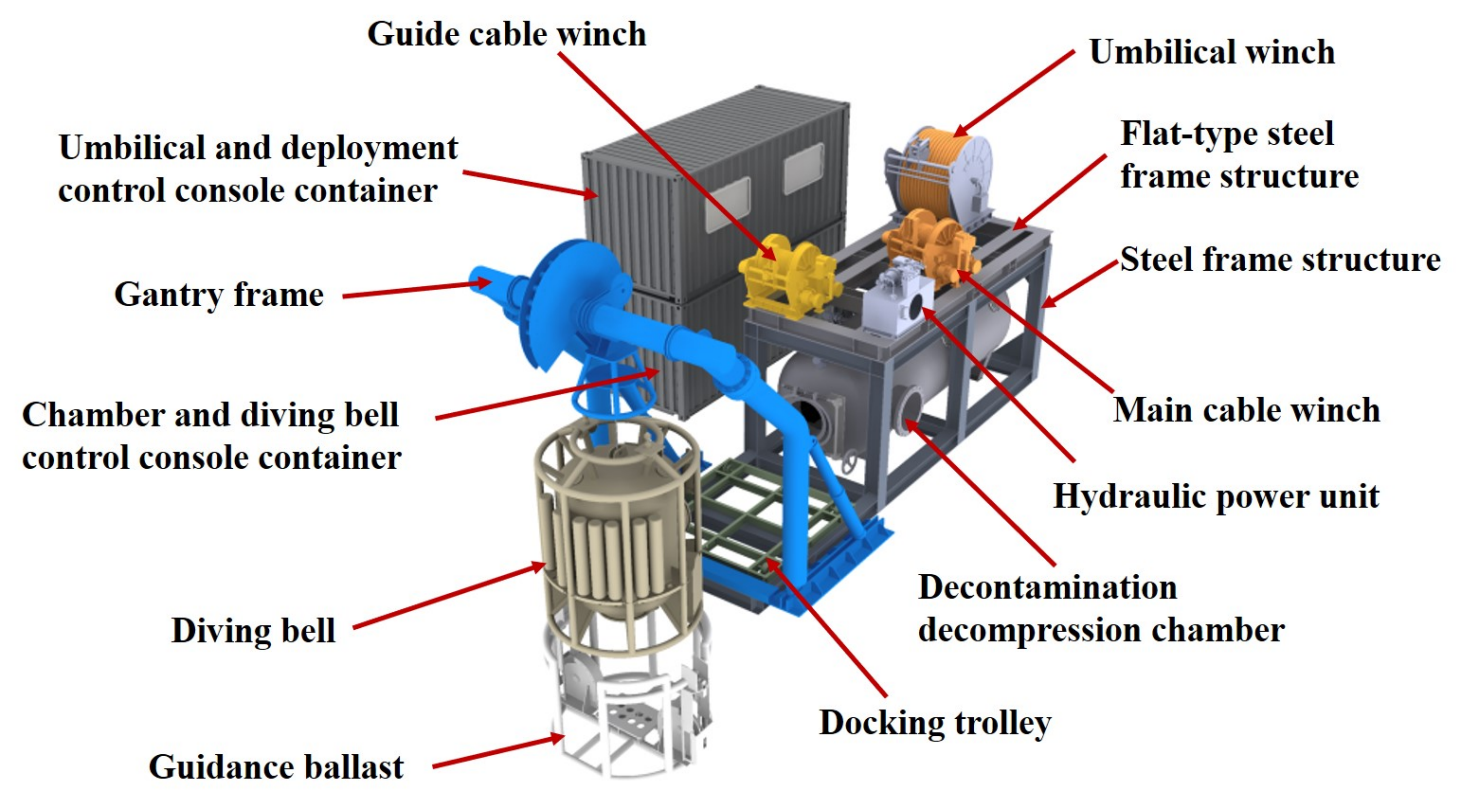
Schematic diagram of the decontamination decompression chamber system components.

The entire saturation diving decontamination decompression chamber system consists primarily of the decompression chamber, diving bell, decompression chamber control console, diving bell control console, launch and recovery system, umbilical and winch system, and the life support system. The equipment adopts a frame and containerized structure fixed on the ship’s deck. The system occupies a deck area of approximately 12,500 mm ∗ 10,000 mm and has an overall height of approximately 8000 mm. Figure 1 shows the overall system layout.

**Figure 2:**
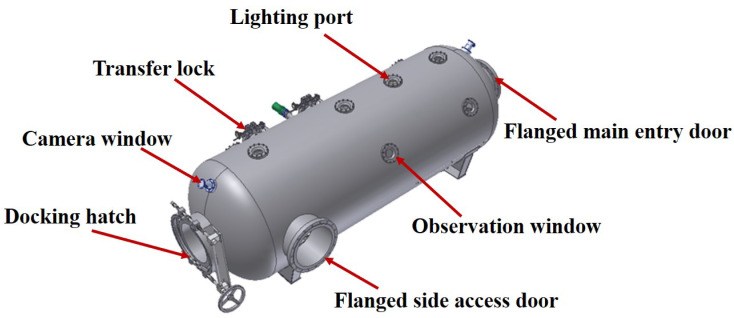
External schematic of the decontamination decompression chamber.

Among the components, the decompression chamber is the core of the system. As shown in Figure 2, its external structure includes the chamber body, hatch, lock, lighting window, camera window, and observation window.

**Figure 3:**
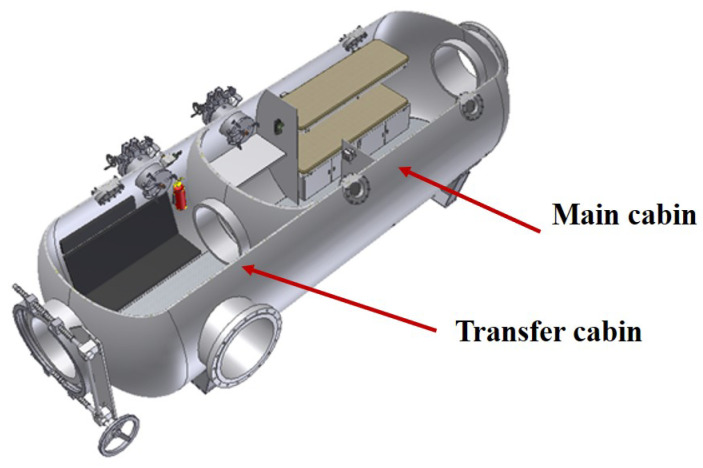
Internal schematic of the decontamination decompression chamber.

Internally, the decompression chamber is composed of a main cabin and a transfer cabin. As illustrated in Figure 3, during actual operation, the diver first enters the transfer cabin for decontamination, which is pressurized to the same level as the diving bell, and then proceeds to the main cabin, where decompression lasting from several hours to several tens of hours is conducted.

The breathing gas supply and exhaust system within the chamber consists of pipelines, valves, instruments, pressure regulators, and gas analyzers, and it provides real-time monitoring of respiratory gas flow. The oxygen supply system must be capable of meeting the maximum flow demand for four persons simultaneously. Exhaled gas from personnel is discharged through an automatic oxygen exhaust regulator. To ensure personnel safety, the oxygen and carbon dioxide concentrations in the cabin environment are strictly regulated. The oxygen concentration should not exceed 23% or fall below 20%. Accordingly, two digital oxygen analyzers are installed on the control console. Sampling pipes from each cabin carry gas to the console, where the flow is regulated via flow meters before entering the gas analyzers. The sample flow is maintained at 300mL/min. For continuous monitoring, the corresponding flow meter is kept at the set flow rate.

### 2.2. Control System Configuration

**Figure 4:**
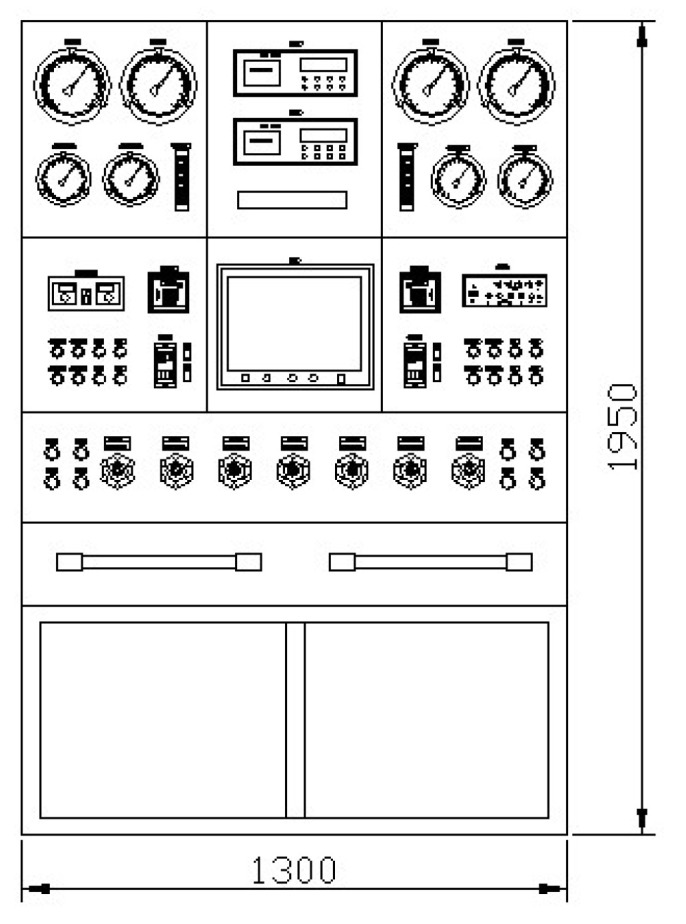
Decompression chamber control console.

As shown in Figure 4, the control console is the primary operation and control unit of the decompression chamber, where all the display instruments and control components are arranged to support centralized control. During system operation, various parameters are monitored in real time using high-precision sensors, including pressure sensors, gas sensors, and valve position feedback sensors. The sensor network covers multiple monitoring points within the chamber. These sensors are connected to data acquisition modules, which convert analog signals into digital signals for transmission to the control system. To ensure data timeliness and reliability, the acquisition module uses a high-speed data acquisition card combined with appropriate signal filtering algorithms to eliminate high-frequency noise and electromagnetic interference, thereby improving data stability and accuracy. Due to factors such as sensor accuracy, acquisition noise, and system latency, raw sensor data may contain anomalies or errors. Before entering the control algorithm, the data undergo cleansing and correction processes, which include outlier removal, interpolation of missing values, and normalization of data with different units. To filter out high-frequency noise from sensor signals, moving average methods are applied to smooth the curves and emphasize slowly varying trends or fault-related features. This preprocessing of multi-sensor data provides support for fault-tolerant control strategies.

**Figure 5:**
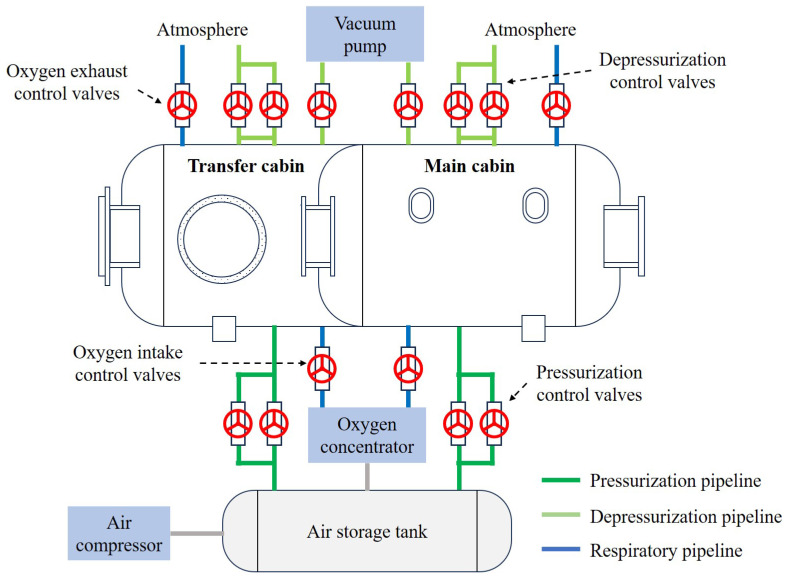
Process flow diagram of the decontamination decompression chamber.

The pressurization and depressurization system represents a central focus of this study as the saturation diving decontamination decompression chamber must support precise pressurization, depressurization, and differential pressure control. The pipeline layout for the pressurization and depressurization system is shown in Figure 5. Both the main cabin and the transition cabin have relatively independent pressurization and depressurization subsystems. The air compressor is connected to an air storage tank that maintains a pressure above 3 MPa. In addition to supplying pressurization gas, the air storage tank provides feed gas to the oxygen concentrator. Each cabin’s pressurization pipeline includes two pipes of different diameters to enable fine adjustment during pressurization. The depressurization pipeline similarly includes two different-sized pipes venting to the atmosphere, and one line connected to the vacuum pump to ensure an effective depressurization rate even when the internal pressure approaches the ambient atmospheric pressure. The piping system also includes oxygen intake and oxygen exhaust pipelines, which influence the overall pressure control. The environmental pressure inside the chamber is maintained through the inlet of the pressurization valves and the outlet of the depressurization valves. However, due to air circulation and ventilation effects, maintaining a stable pressure environment under high-pressure conditions is extremely difficult. Moreover, the pressurization and depressurization valves exhibit slight response delays, leading to significant pressure differentials at the interface between the two chambers. These fluctuations often deviate substantially from the expected pressure values, creating considerable technical challenges for precise control in practice.

During actual operation, the decontamination decompression chamber may encounter various abnormal conditions, including valve calibration offset, valve failure, and chamber leakage, all of which can significantly affect system stability and control accuracy, and even compromise overall safety. Valve calibration offset is often caused by sensor measurement errors, actuator nonlinearities, or mechanical wear over long-term use, resulting in a deviation between the actual control output and the intended target. Traditional control algorithms lack real-time compensation and adjustment capabilities, making them ineffective at handling such deviations and prone to causing response delays or even system instability. Valve failure refers to the loss or degradation of critical valve functions, preventing timely and accurate regulation of key pressure parameters within the chamber, which can lead to control malfunctions and, in severe cases, pose risks to human safety. Chamber leakage is typically caused by structural fatigue, loose connections, or deterioration of sealing components. The static model-based control algorithms commonly used today struggle to detect leakage in real time or respond proactively to developing leak trends. Once leakage occurs, it can quickly escalate, severely threatening the safety of divers and the stable operation of the system.

However, most currently adopted control strategies are based on static models and lack anomaly recognition, fault tolerance, and adaptive adjustment capabilities. These limitations make them inadequate for addressing dynamic changes and fault disturbances in complex operating conditions. In modern complex engineering systems, ensuring reliable operation under dynamic and uncertain conditions has made intelligent data-driven fault diagnosis increasingly important [10]. To overcome these challenges, it is critical to develop a fault-tolerant control algorithm that possesses anomaly detection, auto-tuning, and policy reconfiguration capabilities, thereby enabling the system to effectively respond to dynamic anomalies during decompression chamber operations.

## 3. Reinforced Model-Based Planning Control

The control of the saturation diving decontamination decompression chamber falls under the category of stochastic optimal control problems. We address this by employing a model-based planning approach and optimizing the control process using reinforcement learning techniques.

### 3.1. Model-Based Planning as Control

Stochastic optimal control involves controlling dynamic systems subject to constraints under uncertainty, where such uncertainty affects various components of the system [11]. We formulate the system as a discrete-time dynamic process, expressed as

(1) s(t+1)=f(s(t),a(t),t,w(t),θ)

where s(t) represents the system state, and a(t) denotes the input applied at time *t*. A stochastic phenomenon refers to a process of state transitions, and, when the current state depends solely on the previous state, the process is said to have the Markov property [12]. A stochastic process with the Markov property is referred to as a Markov process. The operation process of the decompression chamber can be regarded as a partially observable Markov decision process (POMDP), where the system state is inferred from observations at different time steps, and the system evolves continuously based on the state transitions and valve operations. The system dynamics are influenced by various uncertainties: θ represents uncertain system parameters modeled as random variables, and w(t) denotes time-varying disturbances or process noise, typically assumed to be independently and identically distributed. Considering the joint uncertainty of the system noise w(t) and model parameters θ, an optimal controller must be designed to handle these challenges. Denoting the observable state as s(t), the control objective is defined through the cumulative reward function:

(2) $$J=E\left(\sum_{t=0}^{T}r\left(s\left(t\right),a\left(t\right),t\right)\right)$$

where E denotes the expectation over all stochastic variables w(t) and θ. The primary challenge of stochastic optimal control lies in satisfying state and input constraints within the closed-loop control horizon. Input constraints typically represent physical limitations of actuators, such as the maximum rate of valve opening changes, while state constraints may take various forms. These can represent physical boundaries of system states, safety-related constraints, or performance requirements associated with desired objectives. Consequently, the resulting constrained stochastic optimal control problem can be formulated as

(3) $$J^{*}=\underset{\left\{\pi_{t}\right\}}{\mathrm{maximize}}E\left(\sum_{t=0}^{T}r\left(s\left(t\right),a\left(t\right),t\right)\right)$$

The constraints include

(4) $$\begin{array}{c} s(t+1)=f(s(t),a(t),t,w(t),\theta )\\ a\left(t\right)=\pi_{t}\left(s\left(0\right),\ldots ,s\left(t\right)\right)\\ \bar{W}=\left[w\left(0\right),\ldots ,w\left(T-1\right)\right]∼Q^{\bar{W}}\\ \mathrm{Pr}\left(\bar{S}=\left[s\left(0\right),\ldots ,s\left(T\right)\right]\right)\geq p_{j}\\ \mathrm{Pr}\left(\bar{A}=\left[a\left(0\right),\ldots ,a\left(T-1\right)\right]\right)\geq p_{j} \end{array}$$

The optimal control policy $\left\{\pi_{t}\right\}$ requires access to all historical state measurements s(t). Once the state transition model of the system environment is established, planning algorithms can be employed to search for the optimal policy. These algorithms operate by selecting actions in the state space, performing forward search, and propagating reward values. Such planning methods require a well-defined state transition model. In the deterministic case, the model specifies the next state for every possible action taken in each state. In stochastic settings, however, it provides a probability distribution over possible next states.

For system identification, we adopt the Context-Aware Dynamics Model (CADM) proposed by Lee et al. [13]. This model enhances generalization across varying environmental dynamics by introducing a context encoder that extracts latent representations of local dynamics and conditions the model predictions accordingly. Furthermore, we apply the PETS (Probabilistic Ensembles with Trajectory Sampling) algorithm, as proposed by Chua et al. [14], which leverages the cross-entropy method (CEM) to optimize the outputs of the learned dynamics model [15]. As the primary focus of this paper is not on system modeling, we do not delve into the specifics of the modeling process. Readers are referred to the original works for a detailed methodology, model architecture, and training procedures.

Model-based planning does not explicitly construct the full policy; instead, it selects the current action based on predicted model outcomes. This approach solves a locally optimal subproblem within a receding horizon to realize control. At each time step, the problem is initialized from the current state $s_{t}$ and solved over a short prediction horizon. Accordingly, the predictive model is defined as

(5) $$s_{i+1|t}=f\left(s_{i|t},a_{i|t},i+t,w_{i|t},\theta \right)$$

where $s_{i|t}$ and $a_{i|t}$ denote the predicted state and action at step *i* within the prediction horizon starting at time *t*, respectively. The initial condition is set as $s_{0|t}=s\left(t\right)$. The predictive dynamics function *f* is typically designed to approximate the true system behavior; however, it may be affected by issues such as insufficient data or model inaccuracy. Despite this, one may proceed without explicitly accounting for model uncertainty and instead rely on the model to perform forward predictions and re-optimize the trajectory at each sampling instant. We define the action sequence as $A=\left[a_{0|t},\ldots ,a_{T-1|t}\right]$, and the objective function is given by

(6) $$J^{*}=\mathrm{maximize}r\left(s_{T|t},a_{T|t},t+T\right)+\sum_{i=0}^{T-1}r\left(s_{i|t},a_{i|t},i+t\right)$$

In model-based planning, when an action is selected at each time step, a set of candidate action sequences is first generated. The expected outcomes of each candidate sequence are then evaluated based on the current state, and the first action of the sequence that yields the best result is selected for execution. Therefore, when applying model-based planning methods, two phases are involved: one is learning the environment model f(s,a) from historical data, and the other is using the learned model to select actions during real-time interactions with the environment. At time step *k*, our objective is to maximize the cumulative reward of the agent. The reward function $r\left(s_{t},a_{t}\right)$ quantifies the reward received by taking action $a_{t}$ in state $s_{t}$. Specifically, the optimization problem is formulated as

(7) $$\underset{a_{k:k+H}}{\mathrm{argmax}}\sum_{t=k}^{k+H}r\left(s_{t},a_{t}\right)\;s.\;t.\;s_{t+1}=f\left(s_{t},a_{t}\right)$$

where *H* denotes the length of the receding horizon, and $\underset{a_{k:k+H}}{\mathrm{argmax}}$ represents the operation of selecting, from all candidate sequences, the action sequence that maximizes the cumulative reward. At each step, we execute the first action $a_{k}$ of the optimal sequence to interact with the environment. One key issue in model-based planning is how to generate candidate action sequences as the quality of these candidates directly affects the quality of the resulting actions. A commonly used method is the cross-entropy method, which we adopt as the baseline algorithm for model-based planning. The detailed steps are presented in Algorithm A1 in Appendix A.1.

### 3.2. Policy-Network-Guided Trajectory Planning

Building upon model-based planning, we propose a policy-network-guided trajectory-planning (PN-TP) method. This approach leverages a policy network trained in conjunction with the environment model to guide trajectory selection during planning. In conventional model predictive control (MPC), the agent uses a system model during online planning but executes only the first action of the planned sequence [16]. In contrast, under the policy-driven control of the PN-TP algorithm, the agent directly executes the action generated by the policy network based on the current observation, similar to standard reinforcement learning paradigms.

In the previous section, the CEM is applied independently at each state to solve the optimization problem. Typically, the sampling distribution is either initialized to a fixed distribution at the beginning of each episode or re-initialized at every time step. However, such initialization schemes suffer from low sampling efficiency due to the lack of a mechanism to generalize valuable information from high-value regions to nearby states. Moreover, information obtained during initialization is discarded afterward, making it difficult to scale the method to high-dimensional solution spaces, which are common in continuous-control environments. To address these limitations, Wang et al. proposed the use of a policy network in CEM-based planning to improve optimization efficiency [17]. They developed two methods: POPLIN-A, which performs optimization in the action space, and POPLIN-P, which performs optimization in the parameter space. POPLIN represents one of the most advanced frameworks for planning from the perspective of policy networks. In our work, we adopt POPLIN as the foundational architecture for the PN-TP algorithm.

In the control process, we denote the action and state spaces by *A* and *S*, respectively. The reward function and state transition function are denoted as $r\left(s_{t},a_{t}\right)$ and $f\left(s_{t+1}|s_{t},a_{t}\right)$, where $s_{t}\in S$ and $a_{t}\in A$ represent the state and action at time step *t*. The agent’s objective is to optimize a policy π by maximizing the expected cumulative reward: J(π)=$E_{\pi }\left[\sum_{t=0}^{\infty }r\left(s_{t},a_{t}\right)\right]$. At time step *i*, the predicted reward for a trajectory $A_{i}=$$\left\{a_{i},a_{i+1},\ldots ,a_{i+\tau }\right\}$ is defined as

(8) $$R\left(s_{i},A_{i}\right)=E\left[\sum_{t=i}^{i+\tau }r\left(s_{t},a_{t}\right)\right]$$

Here, the state sequence $\left\{s_{i},s_{i+1},\ldots ,s_{i+\tau }\right\}$ is obtained by recursively applying the state transition model, such that $s_{t+1}∼f_{\phi }\left(s_{t+1}|s_{t},a_{t}\right)$, indicating that the next state is generated from the current state and action using the transition model $f_{\phi }$. The action sequence $A_{i}$ is generated by the planning module as a candidate trajectory. The expected reward $R\left(s_{i},A_{i}\right)$ is computed by simulating trajectories from the current state using a set of *P* particles. At time step *t*, the *k*-th particle uses the transition model $f_{\phi }^{k,t}\left(s_{t+1}|s_{t},a_{t}\right)$. This model can be constructed from deterministic ensemble models, such as a weighted combination of multiple deterministic models; or probabilistic ensemble models, such as bootstrapped or Bayesian-based transition estimators.

We utilize the policy network to generate a high-quality initial distribution over action sequences, denoted as $\pi \left(s_{t}\right)$. Based on the predicted trajectories provided by the policy network, we inject Gaussian noise into the candidate action sequences and refine them using CEM by optimizing the mean and standard deviation of the noise distribution. This enhances the efficiency of the planning process. Specifically, we define an action sequence as $A_{i}=$$\left\{a_{i},a_{i+1},\ldots ,a_{i+\tau }\right\}$. Similarly, in CEM-based planning, we define a corresponding noise sequence as $A_{i}=\left\{\delta_{i},\delta_{i+1},\ldots ,\delta_{i+\tau }\right\}$. The initial noise distribution is assumed to have a mean $\mu_{0}=0$ and a covariance matrix $\Sigma_{0}=\sigma_{0}^{2}I$, where $\sigma_{0}^{2}$ denotes the initial noise variance. During each CEM iteration, we first select the top ξ action sequences that yield the best performance. The corresponding elite noise sequences are $\left\{\delta_{i}^{0},\delta_{i}^{1},\ldots ,\delta_{i}^{\xi }\right\}$. We then update the parameters of the noise distribution based on these elite samples:

(9) $$\Sigma^{\prime }\leftarrow \mathrm{Cov}\left(\left\{\delta_{i}^{0},\delta_{i}^{1},\ldots ,\delta_{i}^{\xi }\right\}\right),\mu^{\prime }\leftarrow \mathrm{Mean}\left(\left\{\delta_{i}^{0},\delta_{i}^{1},\ldots ,\delta_{i}^{\xi }\right\}\right)$$

Subsequently, we apply a smoothing update scheme to refine the noise distribution. The update formulas are defined as $\mu =\left(1-\alpha \right)\mu +\alpha \mu^{\prime },\Sigma =\left(1-\alpha \right)\Sigma +\alpha \Sigma^{\prime }$, where α is the smoothing parameter that controls the weighting between the old and new distributions. Specifically, at each time step, the CEM procedure is executed for multiple iterations. Candidate actions are resampled, and the noise distribution is updated to complete the trajectory planning. At time step *i*, given an observed state $s_{i}$, a reference action sequence ${\hat{A}}_{i}=\left\{{\hat{a}}_{i},{\hat{a}}_{i+1},\ldots ,{\hat{a}}_{i+\tau }\right\}$ is generated through forward propagation using the policy network. At each planning time step t∈[i,i+τ], the executed action is jointly determined by the policy and the optimized noise, i.e., ${\hat{a}}_{t}=\pi \left({\hat{s}}_{t}\right)$, where ${\hat{s}}_{t}=f_{\phi }\left({\hat{s}}_{t-1},a_{t-1}\right)$ and ${\hat{s}}_{i}=s_{i}$. Given the search noise sequence $\Delta_{i}$, the expected reward is re-estimated to efficiently guide the distribution toward optimal actions. The expected cumulative reward is expressed as

(10) $$R\left(s_{i},\Delta_{i}\right)=E\left[\sum_{t=i}^{i+\tau }r\left(s_{t},\pi \left(s_{t}\right)+\delta_{t}\right)\right]$$

The state transitions are determined by $s_{t+1}=f_{\phi }\left(s_{t+1}|s_{t},\pi \left(s_{t}\right)+\delta_{t}\right)$.

In addition to injecting noise into the action space, we can also introduce noise into the parameter space of the policy network to further enhance the flexibility and efficiency of trajectory planning. Specifically, we denote the parameter vector of the policy network as θ. Starting from time step *i*, we define the parameter noise sequence for the policy as $\Omega_{i}=$$\left\{\omega_{i},\omega_{i+1},\ldots ,\omega_{i+\tau }\right\}$. Under parameter space noise, the expected cumulative reward is defined as

(11) $$R\left(s_{i},\Omega_{i}\right)=E\left[\sum_{t=i}^{i+\tau }r\left(s_{t},\pi_{\theta +\omega_{t}}\left(s_{t}\right)\right)\right]$$

where the state transition is determined by $s_{t+1}=f_{\phi }\left(s_{t+1}|s_{t},\pi_{\theta +\omega_{t}}\left(s_{t}\right)\right)$. Similar to the method applied in the action space, we utilize CEM to optimize the distribution of parameter noise. Specifically, we select the top ξ parameter noise sequences with the highest performance in the current generation: $\left\{\Omega_{i}^{0},\Omega_{i}^{1},\ldots ,\Omega_{i}^{\xi }\right\}$. The noise distribution is then updated based on these elite samples.

(12) $$\Sigma^{\prime }\leftarrow \mathrm{Cov}\left(\left\{\Omega_{i}^{0},\Omega_{i}^{1},\ldots ,\Omega_{i}^{\xi }\right\}\right),\mu^{\prime }\leftarrow \mathrm{Mean}\left(\left\{\Omega_{i}^{0},\Omega_{i}^{1},\ldots ,\Omega_{i}^{\xi }\right\}\right)$$

To simplify the optimization process, we constrain the parameter noise within a single trajectory to remain constant; i.e., $\Omega_{i}=\Omega_{i+1}=\ldots =\Omega_{i+\tau }$. We further propose a method based on policy ensemble learning to efficiently estimate the sampling distribution used in CEM. This approach can be applied to both the action space and the parameter space of the policy network. We define two variants accordingly: PN-TP-A: planning is conducted in the action space; PN-TP-P: planning is conducted in the parameter space of the policy network. Specifically, the planning process involves *M* parallel CEM instances executed simultaneously. For each instance with index i∈{1,…,M}, we denote the sampling distribution parameter as $\psi_{i}$. Then, we define for action-space optimization (PN-TP-A): $\psi_{i}=\phi_{i}$ and for parameter-space optimization (PN-TP-P): $\psi_{i}=\theta_{i}$.

The algorithmic workflow of PN-TP-A can be further specified as follows: in each CEM instance, the policy network takes the current state $s_{t}$ as input and outputs the sampling distribution parameters $\phi_{i}$ for the action space used by that CEM instance. By leveraging the policy network to guide the CEM method in both action and parameter spaces, we can achieve more efficient trajectory planning and action optimization, especially in high-dimensional continuous control problems where this method demonstrates superior performance. The detailed procedure of the PN-TP-A algorithm is presented in Algorithm A2 in Appendix A.2.

The PN-TP-P algorithm performs planning in the parameter space of the policy network. Each policy network outputs its parameter vector $\theta_{i}$. The initial sampling distribution for the *i*-th CEM instance is a Gaussian distribution defined over the policy parameter space, with the mean initialized as $\theta_{i}$. In the argmax operation, the sample $\tau_{i,j}$ denotes the *j*-th sampled parameter vector from the distribution. Its corresponding value is approximated based on the predicted trajectory reward using the learned dynamics model. In contrast to optimization in the action space, which could suffer from non-convexity and local maxima, optimizing over the parameter space allows for structured exploration by injecting noise directly into policy parameters. By employing a stochastic policy network, it naturally encourages broader trajectory exploration and supports re-parameterization within the optimization process. The detailed procedure of the PN-TP-P algorithm is provided in Algorithm A3 in Appendix A.3.

## 4. Meta-Learned Fault-Tolerant Control

To address the fault-tolerant control challenge in saturation diving decontamination decompression chambers under abnormal conditions, this paper proposes a meta-learning-based auto-tuning fault-tolerant control method built upon model-based planning. The proposed approach aims to significantly enhance the robustness and adaptability of the decompression chamber system under complex and abnormal scenarios.

### 4.1. Meta-Learning Based on Memory Inference

Meta-learning, or “learning to learn”, enables models to rapidly generalize to new tasks by extracting common strategies or initial parameters across multiple tasks, thus addressing data scarcity and frequent task-switching scenarios [18]. It involves two nested loops: an inner loop for task-specific adaptation and an outer loop that optimizes meta-level parameters, allowing models to effectively leverage past experiences to solve new problems [19]. Meta-reinforcement learning (meta-RL) generally falls into gradient-based and memory-based inference methods. The gradient-based methods optimize initial model parameters for quick task adaptation but require intensive environment interactions, limiting online efficiency. The memory-based methods directly approximate the Bayes-optimal policy, rapidly converging toward optimal decisions within known task distributions. Considering the real-time interaction constraints and safety requirements in saturation diving decompression chambers, this study adopts a memory-based inference approach for enhanced data efficiency.

In meta-RL, task inference identifies the control process faced by an agent within the inner loop, modeling the agent’s belief about task identity as a posterior distribution over possible tasks [20]. This inferred task distribution guides a base policy, effectively reducing task uncertainty through structured exploration, thus simplifying the meta-learning process into a multi-task learning framework [21]. Within the meta-reinforcement-learning framework, the historical data we use includes all states, actions, and rewards encountered by the policy throughout the entire trial. This data structure enables the agent to learn an adaptive strategy over time. Unlike conventional reinforcement learning, which optimizes the return within a single episode, meta-RL aims to maximize the expected return across an entire trial spanning multiple episodes. The objective is defined as

(13) $$J\left(\theta \right)=E_{M^{i}∼p\left(M\right)}\left[E_{\tau ∼p\left(\tau |M^{i},\theta \right)}\left[\sum_{t=0}^{HT}\gamma^{t}r_{t}\right]\right]$$

where $M^{i}$ denotes a specific task sampled from the task distribution p(M); τ represents the trajectory generated under task $M^{i}$ with meta-policy parameters θ;H is the length of each episode; *T* is the number of episodes per trial; γ is the reward discount factor; $r_{t}$ is the reward at time step *t*.

To optimize the meta-RL objective, memory-based inference treats the entire trial as a continuous sequence without resetting the latent state across episodes. The meta-learned parameters θ are processed through an inference network $f_{\theta }$ to encode memory and reason over time. The task-specific latent state ϕ evolves temporally, updating at each step based on the agent’s interaction history. The detailed procedure of the meta-RL algorithm based on memory-based inference is described in Algorithm A4 in Appendix A.4.

### 4.2. Adaptive Control with Policy Update

Building upon the PN-TP algorithm, we further propose a novel policy-update-based fault-tolerant control (PU-FC) method for auto-tuning fault-tolerant control. This algorithm is specifically designed to enable fast and adaptive fault handling. In this context, sample efficiency is a critical factor, both in terms of the number of samples required from prior experience (meta-training efficiency) and the number of samples needed for adaptation in new tasks (adaptation efficiency). This requirement is particularly pronounced under sparse reward settings, where adaptation efficiency demands that the agent performs inference over task uncertainty. To address this, we leverage probabilistic latent representations learned from prior experiences to model task uncertainty. As the underlying architecture, we adopt PEARL (Probabilistic Embeddings for Actor–Critic Reinforcement Learning), a state-of-the-art off-policy meta-RL algorithm known for its superior performance in handling sparse rewards and task variability [22].

The PEARL framework operates in a “model-free” manner. PU-FC extends it by introducing “model-based” trajectory planning when predefined abnormal events are detected. The algorithm triggers detailed trajectory planning with probability ε to generate high-performance control data under such events for learning. Predefined events refer to boundary conditions in the control system, such as the pressure difference approaching 0 or 200 Pa, or the oxygen concentration approaching 20% or 23%. A system model is built using Gaussian process regression to perform trajectory rollouts. Specifically, the training data for Gaussian process regression is sampled from past $N_{GP}$ time steps of system trajectories, and the future states are predicted as ${\hat{s}}_{t+k}^{d}∼N\left(\mu_{t+k},\Sigma_{t+k}\right)$, where $\mu_{t}\in R^{n}$ and $\Sigma_{t}\in R^{n\times n}$ represent the mean and covariance of predicted system states, estimated based on current and previous observations. To manage computational complexity, we train a separate GP model for each type of abnormal condition, enabling efficient trajectory prediction using GP model under specific dynamics. If no predefined event is detected, PU-FC falls back to the standard PEARL execution mode, selecting actions based on the current state and the inferred latent variable *z*, i.e., $\pi_{\theta }\left(\cdot |s,z\right)$. We empirically set the event-triggering threshold to 0.5. This threshold avoids frequent planning as purely model-based control can reduce action diversity. The overall meta-training procedure of the PU-FC algorithm is detailed in Algorithm A5 in Appendix A.5.

The input consists of a set of training tasks ${\left\{T^{i}\right\}}_{i=1}^{N_{\mathrm{task}\;}}$ sampled from the task distribution p(T). Each training iteration is divided into a data collection phase and a parameter update phase. In the data collection phase, for each sampled task $T^{i}$, the agent collects state transition samples to construct a task-specific replay buffer $H^{i}$. This buffer $H^{i}$ contains trajectories generated by the policy $\pi_{\theta }$ and the context encoder $q_{\phi }$, which was trained during the previous iteration. The latent context variable $u^{i}$ is used as a conditioning input for action generation, guiding the agent’s behavior. During the update phase, the model samples from the replay buffer and updates both the policy network parameters and the context encoder parameters via meta-learning, enabling the agent to rapidly adapt to new tasks or abnormal events.

To enhance the performance and generalization capability of the PU-FC algorithm, at the end of each episode, a context variable $c^{i}$ is sampled from the task-specific replay buffer $H^{i}$, and a new latent context variable $u^{i}$ is drawn from the posterior distribution $q_{\phi }\left(\cdot |c^{i}\right)$. This process is repeated across all tasks to progressively construct a richer replay buffer. During the training phase, for each task $T^{i}$, a context variable $c^{i}$ is independently sampled from $H^{i}$ and used to construct a reinforcement learning mini-batch: $B^{i}={\left\{\left(s^{\left(m\right)},a^{\left(m\right)},c^{\left(m\right)},s^{\prime (m)}\right)\right\}}_{m=1}^{M}$ A latent variable $u^{i}$ is then sampled from $q_{\phi }\left(\cdot |c^{i}\right)$ and used as an external conditioning input for the policy network to generate actions. Training is performed using the Soft Actor–Critic (SAC) framework. The actor loss function is defined as

(14) $$L_{\theta }^{i}=\frac{1}{M}\sum_{m=1}^{M}D_{KL}\left(\pi_{\theta }\left(a|s^{\left(m\right)},u^{i}\right)∥\frac{\exp\left(Q_{\psi }\left(s^{\left(m\right)},a,u^{i}\right)\right)}{U_{\psi }\left(s^{\left(m\right)}\right)}\right)$$

The critic loss function is

(15) $$L_{\psi }^{i}=\frac{1}{M}\sum_{m=1}^{M}{\left[c^{\left(m\right)}+\gamma \bar{V}\left(s^{\prime (m)},u^{i}\right)-Q_{\psi }\left(s^{\left(m\right)},a^{\left(m\right)},u^{i}\right)\right]}^{2}$$

where $\bar{V}$ is the target value function, used to reduce the temporal-difference error of the *Q* function. The loss function for the context feature encoder is defined as

(16) $$L_{\phi }^{i}=L_{\psi }^{i}+\beta D_{KL}\left(q_{\phi }\left(u|c^{i}\right)∥N\left(0,I\right)\right)$$

This loss function adopts a standard Gaussian prior for inference to encourage the context encoder to efficiently learn meaningful context features. Since the critic loss term $L_{\psi }^{i}$ directly affects the approximation quality of the optimal *Q* function, it is incorporated as an additional term in the encoder’s loss. This facilitates the encoder in efficiently identifying specific tasks. During training, the parameters—including the actor policy parameters θ, the critic network parameters ψ, and the context encoder parameters ϕ—are updated using standard stochastic gradient descent (SGD) to achieve efficient convergence.

The meta-test phase applies to newly sampled tasks *T* from the task distribution. As illustrated in Algorithm A6 in Appendix A.6, unlike the meta-training phase, the meta-test phase does not generate actions directly from the policy network. Instead, it executes explicitly planned trajectories. This is because the meta-training process incorporates randomized trajectory planning, which may yield actions that are suboptimal for tasks requiring precise planning. In contrast, meta-testing performs explicit trajectory planning, avoiding potential policy–action mismatches while also reducing computational overhead through simplified planning functions. Specifically, in online meta-tests, the agent performs forward inference using a lightweight trajectory planner without requiring costly optimization at each time step. Similar to the training phase, the posterior distribution of the latent context variable $u^{T}$ is updated at every planning step $t_{\mathrm{update}\;}$ based on newly collected transition data. Upon receiving new transitions, the agent samples a new context vector from the encoder $q_{\phi }$, allowing it to identify the current task or abnormal condition in real time.

## 5. Evaluation Results

In this chapter, we evaluate the performance of the reinforced model-based planning control and meta-learned fault-tolerant control algorithms.

The lower-level control system adopts the Siemens SIMATIC S7-1513R (Munich, Germany), which features a redundancy mechanism to ensure continuous and stable operation. Its core task is to collect data from various sensors and upload it to the upper-level controller while also receiving control commands from the upper-level system and executing corresponding actions. The upper-level controller is powered by an Intel Core i9-12900K processor (Santa Clara, CA, USA), and, to accelerate the execution of control strategies, it is additionally equipped with an NVIDIA GeForce RTX 3090 GPU (Santa Clara, CA, USA). Moreover, to protect the valves, a minimum interval of 1 s is maintained between successive control commands. The PID algorithm is implemented on the PLC with a computation time of less than 5 ms, and, if executed on the upper-level computer, the computation time is less than 1 ms. The average computation time for the model-based MPC algorithm with CEM is 322 ms, whereas the average computation time for the algorithm proposed in this paper is 409 ms. Although the proposed method incorporates meta-learning updates, the use of a policy network to guide the trajectory planning direction helps to reduce the planning budget and thus limits the computation time increase compared to the MPC algorithm based on CEM search.

The system’s control inputs include the opening degrees of four pressurization control valves, two oxygen intake valves, six depressurization valves, two oxygen exhaust valves, and the pressure of the air storage tank. The controlled targets consist of the pressure and oxygen concentration in both the main cabin and the transfer cabin. Based on the target profiles defined by the operation curve, the system regulates both absolute and differential pressure between cabins to reach the desired operational state. Under a maximum working pressure of 1 MPa, it ensures that, during the entire process, whether the inter-cabin door is closed, being opened, or fully open, the pressure in the main cabin remains higher than that in the transfer cabin, with the pressure differential not exceeding 200 Pa. The oxygen concentration is regulated around 21%, with upper and lower bounds of 23% and 20%, respectively. During the execution of the decompression program, the system undergoes a continuous pressure reduction from the equivalent of a 100-meter depth to sea-level pressure. This process ensures that the performance of the control algorithm is evaluated across the full operating pressure range.

We define the reward function as a weighted sum of multiple subitems, where each subitem penalizes the deviation from a specific control objective. Let $p_{m}$ and $p_{t}$ represent the current pressures in the main cabin and transfer cabin, respectively, while $p_{m}^{\mathrm{target}\;}$ and $p_{t}^{\mathrm{target}\;}$ denote their corresponding target pressure trajectories. Let $c_{m}$ and $c_{t}$ denote the current oxygen concentrations in the main and transfer cabins. The reward function is defined as

(17) $$\begin{array}{cc} r= & -\lambda_{1}\left({\left(p_{m}-p_{m}^{\mathrm{target}\;}\right)}^{2}+{\left(p_{t}-p_{t}^{\mathrm{target}\;}\right)}^{2}\right)\\ \; & -\lambda_{2}\cdot \max{\left(0,\Delta p-200\right)}^{2}\\ \; & -\lambda_{3}\cdot I\left(p_{m}<p_{t}\right)\cdot {\left(\Delta p\right)}^{2}\\ \; & -\lambda_{4}\left(I\left(c_{m}<0.20\vee c_{m}>0.23\right)+I\left(c_{t}<0.20\vee c_{t}>0.23\right)\right) \end{array}$$

where $\lambda_{1}$ is defined as coefficient for absolute pressure deviation, $\lambda_{2}$ as coefficient for pressure difference deviation, $\lambda_{3}$ for pressure reversal violation, and $\lambda_{4}$ for oxygen concentration violation. Coefficients $\lambda_{1}$ to $\lambda_{4}$ are assigned based on the safety relevance of each term: $\lambda_{1}=1$, representing general absolute pressure deviation as a baseline; $\lambda_{2}=2$, assigned to pressure differences exceeding the threshold (200 Pa), which may prevent manual opening of the inter-chamber doors and is considered a general safety concern; $\lambda_{3}=8$, reflecting reversed pressure difference between chambers, which poses severe safety risks and must be strongly penalized; and $\lambda_{4}=8$, representing violations in oxygen concentration levels, which are critical to life safety. These coefficients are selected based on the impact of such violations, reflecting the empirical preference that such undesirable deviations should be avoided.

During system operation, the following pressure protection mechanisms are in place: (1) If the deviation between the current pressure and the target pressure exceeds 1000 Pa during operation, the system will pause the running program and adjust the pressure back to the target value. If this adjustment fails within a certain time limit, the system will notify that automatic control is deactivated and switch to manual valve control. During the training process, this protection is set not to be triggered. (2) The decompression chambers are equipped with mechanical safety mechanisms to provide overpressure protection. If the pressure exceeds the maximum allowable range, the device will automatically release to ensure that the internal pressure does not exceed safe limits.

We evaluate the performance of the planning control and fault-tolerant control algorithms from multiple perspectives, including cumulative score, learning efficiency, and performance distribution. For cumulative score, the evaluation metrics include the median, mean, interquartile mean, and optimality gap. The interquartile mean refers to the average score computed after discarding the lowest 25% and highest 25% of all the trial results [23]. The optimality gap measures the deviation between the algorithm’s achieved score and the theoretical optimum, reflecting the algorithm’s ability to approach the optimal solution. Learning efficiency focuses on the trend of the objective function during training; by observing the convergence rate and fluctuations, we can analyze how efficiently the algorithm utilizes data and how dynamically it approaches optimal performance. The performance distributions present the score distributions across multiple runs, highlighting the consistency and stability of the algorithm’s output.

### 5.1. Performance Evaluation of Trajectory Planning

The compared methods include the classic PID-based control algorithm, the method that uses the CADM model combined with the cross-entropy method for model output optimization (denoted as CEM), the POPLIN framework proposed by Wang and Ba, specifically POPLIN-A (optimization in the action space) and POPLIN-P (optimization in the action space), as well as our proposed policy-network-guided trajectory planning methods (PN-TP), including both PN-TP-A and PN-TP-P.

To eliminate the influence of different neural network architectures, all the algorithms are implemented based on the SAC (Soft Actor–Critic) framework. The hyperparameter settings of the SAC algorithm are summarized in Table A1. A hyperparameter search is conducted for the baseline method POPLIN, and the configuration yielding the best performance is selected. The same hyperparameter settings are subsequently adopted in the PN-TP method to demonstrate its performance improvement relative to POPLIN.

**Figure 6:**
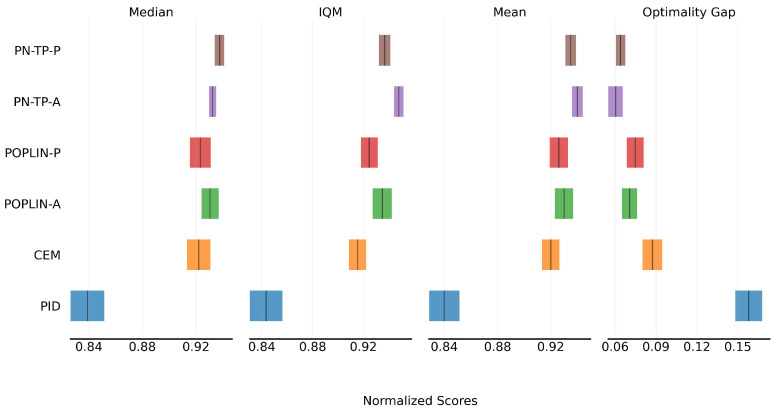
Cumulative score comparison of control algorithms.

Figure 6 shows the cumulative score of the algorithms. From the four metrics, it is evident that the incorporation of policy-network guidance leads to improved performance for model-based planning control. Compared to the baseline CEM method, POPLIN-A and POPLIN-P achieve performance gains of 1.0% and 0.6%, respectively. Notably, POPLIN-A, which performs optimization in the action space, outperforms POPLIN-P, which optimizes in the parameter space. This may be attributed to the fact that model-based planning algorithms inherently operate in the action space, making action-space optimization more direct and effective. The proposed PN-TP-A and PN-TP-P further improve upon the baseline POPLIN methods, achieving approximately 1.1% and 0.9% performance gains, respectively. Among them, PN-TP-A delivers the best performance, with an overall improvement of approximately 9.6% compared to the conventional PID control method.

**Figure 7:**
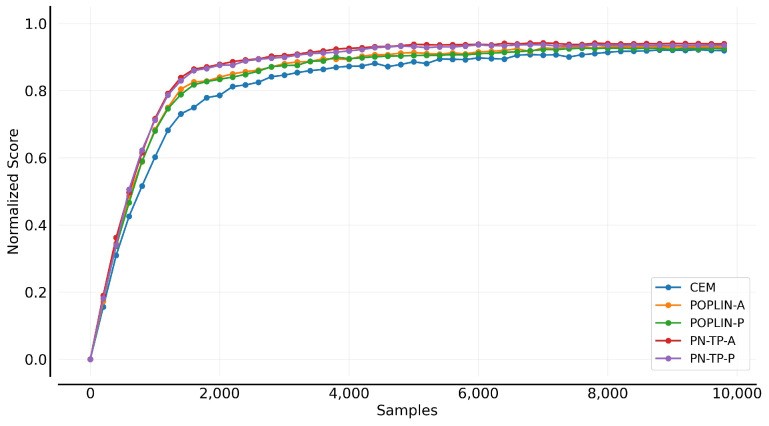
Learning efficiency comparison of control algorithms.

Figure 7 compares the progressive and convergent performance of the model-based planning control algorithms. It is observed that the use of policy-network guidance significantly improves both early-stage progress and final convergence compared to the baseline CEM algorithm. Specifically, POPLIN-A, which optimizes deterministic action sequences based on the initial state distribution, and POPLIN-P, which injects noise into the policy network, both demonstrate faster learning progression. The proposed PN-TP-A and PN-TP-P show further improvements over POPLIN-A and POPLIN-P in terms of learning progression, especially within the first 4000 time steps, enabling the agent to adapt more quickly to the environment and optimize control decisions. Moreover, both methods converge to a stable state in the later stages of training.

**Figure 8:**
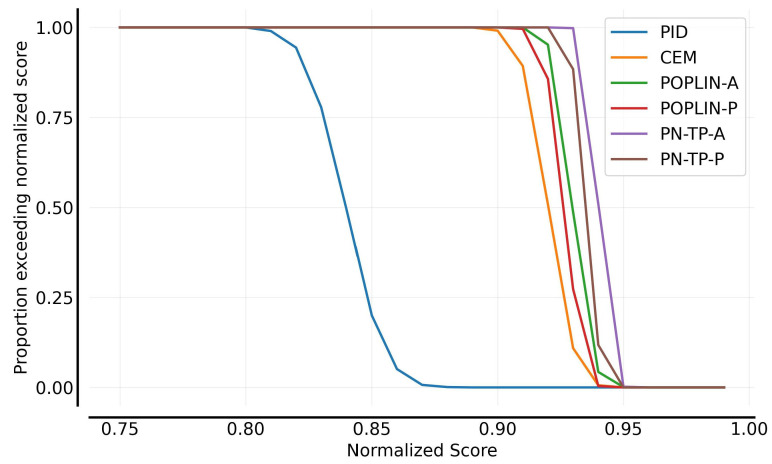
Performance distribution comparison of control algorithms.

Figure 8 illustrates the performance distributions of the planning algorithms. Compared to PID-based control, all the model-based planning algorithms demonstrate significantly superior performance. Policy-network-guided planning algorithms, including POPLIN-A, POPLIN-P, PN-TP-A, and PN-TP-P, leverage historical experience to predict better actions, which enhances adaptability to environmental changes. As a result, these algorithms exhibit more favorable overall performance distributions, substantially outperforming the PID control approach.

### 5.2. Performance Evaluation of Fault-Tolerant Control

In the fault-tolerant control experiments, three types of abnormal conditions are considered: (1) valve calibration offset, simulated by introducing small random perturbations to the valve calibration value; (2) valve failure, simulated by setting a valve to remain fully closed or fixed at a specific opening, regardless of the input action, while either correctly or incorrectly reporting execution of the intended control commands to the system; and (3) chamber leakage, simulated by introducing additional unintended valve openings.

More precisely, the above abnormal conditions can be detailed regarding the following six cases:

1. Valve calibration offset: The valve’s actuation threshold drifts over time. For example, the valve used to begin opening at a 5% control input but now only starts to open at 6% after a period of use.
2. Valve failure with true feedback: The valve remains fully closed regardless of the control input and correctly reports its closed state to the control system.
3. Valve failure with false feedback: The valve remains fully closed regardless of the input but falsely reports to the control system that it is operating at the commanded opening level.
4. Fixed valve opening with true feedback: The valve stays open at a fixed random position between 2% and 10%, regardless of the input, and reports this fixed opening accurately to the control system.
5. Fixed valve opening with false feedback: The valve remains open at a fixed random position between 2% and 10%, regardless of the input, but falsely reports that it is operating at the commanded opening level.
6. Simulated leakage through additive opening: The valve is opened at a fixed level between 1% and 5%, and any control input is additively applied on top of this value to simulate leakage in the chamber.

All of the above scenarios are implemented by modifying the final command sent from the control system to the valve, thereby simulating fault conditions.

We use the best-performing policy-network-guided action-space planning algorithm, PN-TP-A, from the previous chapter as a baseline, which does not incorporate meta-learning. The probabilistic-embedding-based meta-reinforcement learning method used for comparison is denoted as PEARL, and the proposed algorithm is denoted as PU-FC. The hyperparameter settings for the algorithms are listed in Table A2. Similarly, a hyperparameter search is conducted for the baseline method PEARL, and the same hyperparameter settings are subsequently adopted in the PU-FC method.

Table 1 presents the averaged cumulative score comparison. Across all the evaluation metrics, meta-learning-based methods PEARL and PU-FC outperform both the PID-based controller and the previously best-performing PN-TP-A. The traditional PID method exhibits a significant performance drop when facing fault conditions involving inconsistent information or deceptive feedback signals. The PN-TP-A algorithm achieves certain improvements over PID; however, it still shows limitations under fault scenarios. PEARL and PU-FC achieve approximately 8.8% and 9.7% improvements over PN-TP-A, respectively. This advantage primarily stems from the enhanced generalization capability and task-adaptive optimization of the meta-learning framework, enabling the agent to achieve more stable and adaptive decision-making in a wider range of task environments. In particular, for false-feedback faults, such as case 3 and case 5, PU-FC achieves notable performance improvement compared to PEARL, demonstrating its superior fault tolerance against misleading system state estimations. PU-FC incorporates policy learning for fine-grained planning under abnormal events, allowing for more accurate modeling of state distribution characteristics and leading to improved performance over PEARL.

In rare cases, multiple exceptions may occur simultaneously. To evaluate scenarios where multiple faults occur simultaneously, we selected three representative combinations of fault cases: (1) case 1–2–4, simulating actuation deviation along with faults under truthful feedback; (2) case 1–3–5, combining deviation with two types of deceptive feedback; and (3) case 2–3–6, involving both truthful and deceptive feedback under fault conditions, along with simulated leakage. Under these compound fault disturbances, all the algorithms experience performance degradation. Nevertheless, as shown in Table 2, PU-FC consistently achieves the highest cumulative scores across all the combinations, demonstrating its robustness and adaptability in multi-fault conditions.

During operation, we expect the algorithm to consistently achieve accurate process control under all the conditions without necessarily identifying the specific type of fault. Prior to each actual run of the decompression chamber, a thorough inspection is conducted for each unit, and any detected fault is resolved at that stage.

**Figure 9:**
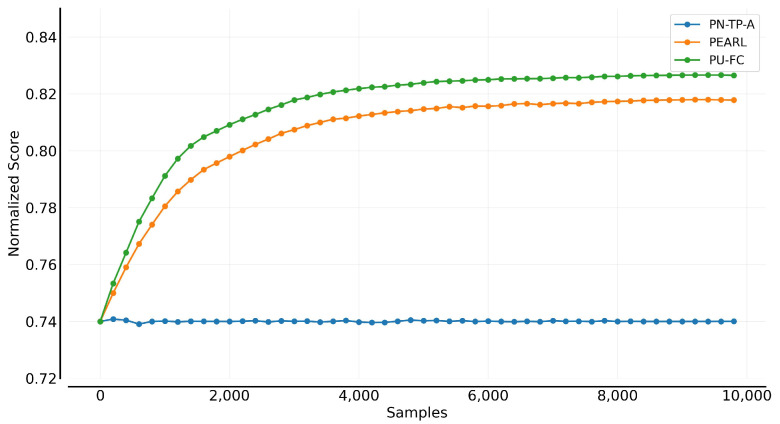
Learning efficiency comparison in fault-tolerant control.

Figure 9 compares the learning progress and convergence performance of the auto-tuning fault-tolerant control algorithms. PU-FC demonstrates better convergence than PEARL, achieving over 80% of its final performance within approximately 2000 training steps. The proposed meta-learning method accelerates policy optimization, enabling the agent to quickly adapt to its environment, thereby ensuring control system efficiency and reliability. During training, PU-FC also maintains more stable learning dynamics, reflected by fewer fluctuations and smoother learning curves.

**Figure 10:**
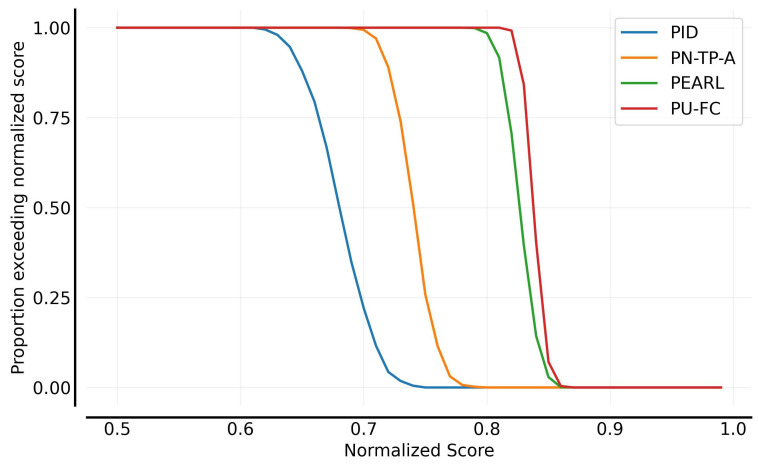
Performance distribution comparison in fault-tolerant control.

Figure 10 shows the performance distributions of the planning algorithms. Compared with PID and the non-meta-learning method PN-TP-A, both PEARL and PU-FC exhibit improved performance distributions. PU-FC demonstrates superior adaptability to environmental variations and reduced decision-making latency, resulting in a more favorable overall performance profile.

We conduct generalization tests for the PU-FC algorithm by evaluating its performance on fault cases excluded from the meta-training task distribution. Specifically, each of the six fault cases is individually excluded during training and then used as a test task after training. The results are summarized in Table 3. Overall, PU-FC exhibits only a minor performance drop of approximately 1.9%, demonstrating strong adaptability to previously unseen fault scenarios. Notably, the performance degradation in cases 2–5 is relatively small, which may be attributed to the underlying similarities among these scenarios. This highlights the PU-FC algorithm’s effective transferability across related fault conditions.

In the context of decompression chamber control, the decompression process must follow a strictly regulated pressure curve that aligns with the physiological tolerance of the human body. Any deviation or fluctuation in pressure, especially under actuator faults, can lead to serious issues, such as discomfort, barotrauma, or decompression sickness, for occupants inside the chamber. The proposed PU-FC algorithm is specifically designed to mitigate such risks by enabling rapid fault adaptation and maintaining precise tracking of the pressure trajectory even in the presence of disturbances. The improvement in performance reflects the controller’s enhanced capability to suppress error oscillations and maintain smooth pressure transitions, which directly contributes to preserving a stable and comfortable decompression environment.

## 6. Concluding Remarks

In this paper, we present a meta-reinforced-model-based planning and fault-tolerant control framework for a saturation diving decontamination decompression chamber. The key contributions of this work are summarized as follows:

- Policy-network-guided trajectory-planning (PN-TP) algorithm: We develop a model-based trajectory-planning algorithm reinforced by an ensemble of policy networks, which improves planning efficiency and consistency under system uncertainty. This method leverages the ensemble of learned policies to prioritize the search for optimal control actions, achieving more reliable trajectories in the face of disturbances.
- Policy-update-based fault-tolerant control (PU-FC) algorithm: We propose a meta-learning fault-tolerant control strategy that incorporates fine-grained action planning near boundary conditions during training and uses rapid policy adaptation to handle system perturbations and faults. By meta-training on various fault scenarios, the controller can auto-tune its parameters, enabling the system to sustain stable operation without manual reconfiguration.
- Control system design and implementation: We conduct a comprehensive design and implementation of a robust control system specifically for the saturation diving decontamination decompression chamber. This work addresses critical control challenges, including high-precision pressure regulation, high stability under varying operational states, and high reliability in the presence of potential actuator faults and environmental disturbances. The successful realization of this advanced control framework demonstrates its effectiveness in meeting stringent performance requirements in practical operations.

In conclusion, the meta-reinforced planning and fault-tolerant control approach developed in this work addresses the control requirements in multi-compartment hyperbaric systems. The combination of model-based planning, reinforcement learning guidance, and meta-learning adaptation provides a powerful and flexible control solution. We have shown that this approach can maintain high precision, stability, and reliability in the face of uncertainties and faults, paving the way for more intelligent life-support system applications beyond saturation diving. The saturation diving decontamination decompression chamber, as a specialized Pressure Vessel for Human Occupancy (PVHO), demonstrates the potential for extending these control methods to a broader range of PVHOs, which are commonly used in aerospace, deep-sea exploration, and high-altitude rescue operations. By employing learning algorithms to identify shared features across different types of pressure chambers, the generalization and adaptability of the algorithms can be further enhanced. This will contribute to the development of a more universal control framework, facilitating its application across a diverse range of PVHOs. Future enhancements in adaptive learning, fault diagnosis, and system integration will further strengthen the capability of such controllers, bringing us closer to fully autonomous and fault-resilient operations of life-critical engineering systems.

## Figures and Tables

**Table 1:** Cumulative score comparison in fault-tolerant control under six cases.

Algorithm	Case						Average
	**1**	**2**	**3**	**4**	**5**	**6**	
PID	0.751	0.662	0.648	0.693	0.667	0.695	0.686
PN-TP-A	0.826	0.739	0.687	0.741	0.702	0.753	0.741
PEARL	0.913	0.815	0.779	0.845	0.786	0.834	0.829
PU-FC	0.920	0.821	0.789	0.846	0.796	0.855	0.838

**Table 2:** Cumulative score comparison in fault-tolerant control under different combinations of cases.

Algorithm	Combinations of Cases		
	**1–2–4**	**1–3–5**	**2–3–6**
PID	0.612	0.569	0.512
PN-TP-A	0.668	0.627	0.549
PEARL	0.743	0.675	0.602
PU-FC	0.753	0.684	0.613

**Table 3:** Test on fault case excluded from the meta-training task.

Algorithm	Case						Average
	**1**	**2**	**3**	**4**	**5**	**6**	
PU-FC	0.892	0.808	0.772	0.832	0.780	0.829	0.819

## Data Availability

Dataset available on request from the authors.

## Appendix A. Algorithms

### Appendix A.1. Algorithm A1

**Algorithm A1:** Model-Based Planning with Cross-Entropy Method	
**Input:** Initialize the sampling distribution P(A) with *N* trajectories using a stochastic policy	
1	for *K* iterations do
2	Sample *N* action sequences $A_{1},\ldots ,A_{N}$ from the distribution P(A)
3	For each action sequence $A_{1},\ldots ,A_{N}$, evaluate the cumulative reward using the environment model
4	Select the top *M* action sequences $A_{i_{1}},\ldots ,A_{i_{M}}$ based on the evaluation results
5	Update the distribution P(A) using the selected top *M* sequences
6	Compute the average of the first actions in the top-performing sequences and execute it as the current action

### Appendix A.2. Algorithm A2

**Algorithm A2:** Policy-Network-Guided Trajectory Planning in Action Space (PN-TP-A)	
**Input:** Initial policy networks $p_{i}$, where $\theta_{i},i=1,2,\ldots ,M$ (represent the parameters of the ensemble policy networks); planning horizon *H*; initial dynamic model $f_{\omega }$ parameterized by ω; empty datasets $D_{m}$ and $D_{p}$	
1	Use a random policy for rollout to populate dataset $D_{m}$, storing tuples $\left\{\left(s_{t},a_{t},s_{t+1}\right)\right\}$
2	Update model parameters ω using dataset $D_{m}$ with supervised loss
3	while not terminated do
4	Set time step t=0, clear planning dataset $D_{p}=\left\{\right\}$
5	for *K* trials do
6	for each policy network $p_{i}$ in the ensemble do
7	Use $p_{i}$ and model $f_{\omega }$ to generate mean action sequence $\mu_{i}$
8	$\mu_{i},v_{i}=\mathrm{CEM}\left(\mu_{i}\right)$
9	${\hat{a}}_{t,i}={\hat{\mu }}_{i}\left[0\right]$
10	Select best action across ensemble $a_{t}={\mathrm{argmax}}_{{\hat{a}}_{t,i}}v_{i}$
11	$s_{t+1}=\mathrm{step}\left(a_{t}\right)$
12	Add transition $\left(s_{t},a_{t},s_{t+1}\right)$ to model dataset $D_{m}$
13	Add planning data ${\left\{\left(s_{t},a_{t,i}\right)\right\}}_{i=1}^{M}$ to dataset $D_{p}$
14	t=t+1
15	Until maximum episode length or terminal state
16	Update model ω using collected dataset $D_{m}$
17	Update policy parameters ${\left\{\theta_{i}\right\}}_{i=1}^{M}$ using planning dataset $D_{p}$ via behavior cloning loss

### Appendix A.3. Algorithm A3

**Algorithm A3:** Policy-Network-Guided Trajectory Planning in Parameter Space (PN-TP-P)	
**Input:** Initial policy networks $p_{i}$, with parameters $\theta_{i},i=1,2,\ldots ,M$ (where *M* is the ensemble size); planning horizon *H*; initial dynamics model $f_{\omega }$ with parameters ω; initialize empty datasets $D_{m}$ and $D_{p}$	
1	Use a random policy for rollout to populate dataset $D_{m}$, storing tuples $\left\{\left(s_{t},a_{t},s_{t+1}\right)\right\}$
2	Use dataset $D_{m}$ to update model parameters ω via mean squared error loss
3	while not terminated do
4	Set time step t=0, clear planning dataset $D_{p}=\left\{\right\}$
5	for *K* trials do
6	for each policy network $p_{i}$ in the ensemble do
7	$\mu_{i},v_{i}=\mathrm{CEM}\left(\theta_{i}\right)$
8	$\delta_{i}=\mu_{i}\left[0\right]$
9	$\theta_{t}={\mathrm{argmax}}_{\theta_{i}+\delta_{i}}v_{i}$
10	$a_{t}=p_{\theta_{t}}\left(s_{t}\right)$
11	$s_{t+1}=\mathrm{step}\left(a_{t}\right)$
12	Add transition $\left(s_{t},a_{t},s_{t+1}\right)$ to dataset $D_{m}$
13	Add the parameter noise data ${\left\{\delta_{i}\right\}}_{i=1}^{M}$ from all policy networks to the dataset $D_{p}$
14	t=t+1
15	until maximum episode length or terminal condition
16	Update model parameters ω using dataset $D_{m}$
17	Update policy parameters ${\left\{\theta_{i}\right\}}_{i=1}^{M}$ using dataset $D_{p}$ via supervised training

### Appendix A.4. Algorithm A4

**Algorithm A4:** Meta-Learning with Memory-Based Inference	
**Input:** Initial meta-parameters θ	
1	while not converged do
2	Sample a task $M^{i}∼p\left(M\right)$ from the task distribution
3	for each task $M^{i}$ do
4	Initialize latent state $\phi_{0}$
5	Execute a multi-episode trial on task $M^{i}$
6	for each time step *t* in the trial do
7	Observe current state $s_{t}$, previous action $a_{t-1}$, and previous reward $r_{t-1}$
8	Update latent state: $\phi_{t}=f_{\theta }\left(s_{t},a_{t-1},r_{t-1},\phi_{t-1}\right)$
9	Sample action $a_{t}∼\pi_{\theta }\left(\cdot |s_{t},\phi_{t}\right)$
10	Execute action $a_{t}$ in the environment and receive reward $r_{t}$
11	Update meta-policy parameters θ using the total discounted return over the entire trial

### Appendix A.5. Algorithm A5

**Algorithm A5:** Meta-Training Algorithm for Policy-Update-Based Fault-Tolerant Control (PU-FC)	
**Input:** Training task set ${\left\{T^{i}\right\}}_{i=1}^{N_{\mathrm{task}}}∼p\left(\cdot \right)$	
1	Initialize replay buffers $H^{\left(i\right)}$ and network parameters ϕ,θ,ψ
2	for iteration =1,2,… do
3	for each task $T^{i}$ do
4	Initialize context set $c^{i}=\left\{\right\}$; sample latent variable $u^{i}∼N\left(0,I\right)$
5	for episode =1 to *E* do
6	for time step t=0 to T−1 do
7	if abnormal event occurs and rand ≤ε, then
8	Construct Gaussian process regression dataset: $D_{t}={\left\{\left(s_{t-l}^{d},s_{t-l+1}^{d},\ldots ,s_{t}^{d}\right)\right\}}_{l=1}^{N_{GP}}$
9	Perform GP-based trajectory prediction: ${\left\{{\hat{s}}_{t+k}^{d}\right\}}_{k=1}^{K}$
10	Perform trajectory planning based on predictions from the GP model to derive control actions $a_{t}$
11	else
12	Sample action from policy: $a_{t}∼\pi_{\theta }\left(\cdot |s_{t},u^{i}\right)$
13	Apply $a_{t}$; observe $\left(s_{t},a_{t},c_{t},s_{t+1}\right)$
14	Add transition $\left(s_{t},a_{t},c_{t},s_{t+1}\right)$ to replay buffer $H^{i}$
15	Update context set:
16	$c^{i}={\left\{(s^{\left(n\right)},a^{\left(n\right)},c^{\left(n\right)},s^{\prime (n)})\right\}}_{n=1}^{N}∼H^{i}$
17	Sample new latent context: $u^{i}∼q_{\phi }\left(\cdot |c^{i}\right)$
18	for l=1 to $N_{\mathrm{train}}$ do
19	for each task $T^{i}$ do
20	Sample $c^{i}∼S_{c}\left(H^{i}\right)$, mini-batch $B^{i}∼H^{i}$
21	Sample $u^{i}∼q_{\phi }\left(\cdot |c^{i}\right)$
22	$\phi \leftarrow \phi -\alpha_{\phi }\nabla_{\phi }\sum_{i}L_{\phi }^{i}$, $\theta \leftarrow \theta -\alpha_{\theta }\nabla_{\theta }\sum_{i}L_{\theta }^{i}$
23	$\psi \leftarrow \psi -\alpha_{\psi }\nabla_{\psi }\sum_{i}L_{\psi }^{i}$

### Appendix A.6. Algorithm A6

**Algorithm A6:** Meta-Testing Algorithm for Policy-Update-Based Fault-Tolerant Control (PU-FC)	
**Input:** Meta-test task T∼p(·)	
1	Initialize context embedding set $c^{T}=\left\{\right\}$
2	for episode =1,2,… do
3	for t=0 to T−1 do
4	if context update trigger is met $\left(t\;\mathrm{mod}\;t_{\mathrm{update}}\equiv 0\right)$, then
5	Sample new latent variable $u^{T}∼q_{\phi }\left(\cdot |c^{T}\right)$ from the context encoder
6	Sample action $a_{t}∼\pi_{\theta }\left(\cdot |s_{t},u^{T}\right)$ using the policy network and current context
7	Execute control $a_{t}$ and observe $\left(s_{t},a_{t},c_{t},s_{t+1}\right)$
8	Update context set $c^{T}\leftarrow c^{T}\cup \left\{(s_{t},a_{t},c_{t},s_{t+1})\right\}$

## Appendix B. Hyperparameters

**Table A1:** SAC hyperparameters.

Hyperparameter	Value
Discount factor	0.99
Random seed	1000
Replay buffer size	Infinite
Sampling type	Uniform sampling
Optimizer	$\mathrm{Adam}\left(\beta_{1}=0.9,\beta_{2}=0.999\right)$
Learning rate	$3\times 10^{-4}$
Batch size	512
Soft update coefficient	0.99
Steps per target network update	1
Target network update frequency	2

**Table A2:** Hyperparameter settings for PEARL and PU-FC.

Hyperparameter	Value
Discount factor	0.99
Initialization steps	5000
Number of stacks	2
Replay buffer size	Infinite
Number of demonstrations	5
Batch size	256
Action repeat	2
Resampling rate	75% → 25%
Planning horizon	1→5
Validation frequency	2000
Number of validation rollouts	30
Number of planning candidates	6
Rollout length	100
Soft update coefficient	0.99
Time discount coefficient	0.5
Loss function weighting	$\alpha_{1}=0.5,\alpha_{2}=0.1,\alpha_{3}=2$
Action exploration noise	0.1→0.05
Optimizer	$\mathrm{Adam}\left(\beta_{1}=0.9,\beta_{2}=0.999\right)$
Learning rate	$3\times 10^{-4}$
MLP hidden units	512×2
MLP activation function	ELU
Latent context embedding dimension	50
Target network update frequency	2
